# Effects of a novel rice-based diabetes-specific formula on postprandial glucose and gastrointestinal hormones: a double-blinded multi-arm randomized crossover trial

**DOI:** 10.3389/fendo.2023.1141497

**Published:** 2023-05-24

**Authors:** Supat Chaiyakul, Narong Ketkham, Chartchai Chaichana, Nanta Khumkhana, Wanjan Deekum, Pakwuan Wongshaya, Thaniya Suwanmalai, Carol Hutchinson, Pornpoj Pramyothin

**Affiliations:** ^1^ Department of Nutrition, Faculty of Public Health, Mahidol University, Bangkok, Thailand; ^2^ Department of Medicine, Faculty of Medicine Siriraj Hospital, Siriraj Hospital, Mahidol University, Bangkok, Thailand

**Keywords:** rice, diabetes, glycemic index, glucose, insulin, GLP-1, medical food, crossover trial

## Abstract

**Introduction:**

We developed a novel rice-based medical food for diabetes (MFDM) powder formula, using locally available ingredients in Thailand, which can potentially improve patient access to diabetes-specific formula (DSF) by reducing cost and improving availability.

**Purpose:**

The goals of our studies were to 1) measure the glycemic index (GI) and glycemic load (GL) of the MFDM powder formula in healthy individuals, and 2) assess postprandial glucose, insulin, satiety, hunger, and gastrointestinal (GI) hormone responses in adults with prediabetes or early type 2 diabetes after consuming MFDM in comparison with a commercially available standard formula (SF) and a DSF.

**Methods:**

In Study 1, glycemic responses were assessed using the area under the curve (AUC), which was used to calculate GI and GL. Study 2 was a double-blinded multi-arm randomized crossover trial enrolling participants with either prediabetes or type 2 diabetes of ≤6 years. At each study visit, participants consumed either MFDM, SF, or DSF which contained 25 g of carbohydrates. Hunger and satiety were assessed using a visual analog scale (VAS). Glucose, insulin, and GI hormones were assessed using AUC.

**Results:**

All participants tolerated the MFDM well with no adverse events. In Study 1, the measured GI was 39 ± 6 (low GI) and GL was 11 ± 2 (medium GL). In Study 2, glucose and insulin responses were significantly lower after MFDM compared with SF (*p-*value<0.01 for both), however, those responses were similar between MFDM and DSF. MFDM suppressed hunger, promoted satiety, stimulated active GLP-1, GIP, and PYY, and suppressed active ghrelin although these changes were similar to SF and DSF.

**Conclusions:**

MFDM had a low GI and a low-to-medium GL. In people with prediabetes or early type 2 diabetes, MFDM elicited reduced glucose and insulin responses when compared with SF. Rice-based MFDM may be an option for patients who are at risk for postprandial hyperglycemia.

**Clinical Trial Registration:**

https://www.thaiclinicaltrials.org/show/TCTR20210731001, identifier TCTR20210731001; https://www.thaiclinicaltrials.org/show/TCTR20210730007, identifier TCTR20210730007.

## Introduction

Diabetes-specific enteral nutrition formulas (DSFs) are an integral part of the management of patients with diabetes. In inpatient settings, oral and enteral nutrition often result in hyperglycemia which has been associated with adverse outcomes including increased mortality (1). The use of DSFs in this setting can reduce the risk of hyperglycemia and improve glucose variability (2). In outpatient settings, DSFs have been successfully used as meal replacements which can lead to improved glycemia, weight loss (3), and diabetes remission (4). In malnourished patients, the use of DSFs has resulted in a lower risk of hospital visits and admissions and reduced healthcare costs (5).

Meta-analyses have shown that DSFs can reduce postprandial glucose responses, improve HbA1c and lipid profile, and promote satiety due to their low glycemic index (GI) and glycemic load (GL), increased fiber and protein content, and the use of healthy lipid blends (6–8). GI represents the postprandial glycemic response to a particular kind of food relative to glucose, while GL represents a similar concept but also takes into account the carbohydrate amount within a serving of such food (9). Previous studies have also shown that DSFs have direct effects on gastrointestinal hormones, namely glucagon-like peptide-1 (GLP-1), glucose-dependent insulinotropic peptide (GIP), peptide YY (PYY), and ghrelin (10, 11). GLP-1 and GIP are known to play an important role in the regulation of postprandial glucose by stimulating insulin secretion (12). Both hormones, in conjunction with PYY, are also known to be important satiety signals, while ghrelin works by stimulating appetite and increasing food intake (13).

Despite its benefits, patient access to DSF remains limited in many clinical settings, particularly in low- and middle-income countries such as Thailand, with cost and lack of reimbursement being important barriers. For this reason, the development of novel DSFs using locally available ingredients can potentially improve patient access to DSFs by reducing cost and improving availability.

Rice (*Oryza sativa* L.) is the main dietary staple for more than half of the world’s population and is grown in more than 100 countries (14). It contains complex carbohydrates, protein, and fat which provide energy and is a good source of dietary fiber, gamma-oryzanol, and phytosterols, which are found mainly in the membranes of rice and germs. Results from previous studies showed that rice bran oil with a high content of gamma-oryzanol reduced blood sugar and lipid levels (15, 16). For this reason, rice was used to develop and manufacture a novel DSF in this study.

The objectives of this study were twofold. The first objective was to measure the glycemic index and glycemic load of a novel rice-based diabetes-specific formula (MFDM, medical food for diabetes mellitus, powder formula) in healthy individuals. The second objective was to compare postprandial responses in glucose, insulin, gastrointestinal hormones, satiety, and hunger after consumption of the MFDM with commercially available formulas, including a non-diabetes-specific standard formula (SF) and a diabetes-specific formula (DSF) in adults with recent-onset diabetes or those who were at risk for diabetes.

## Materials and methods

### Development of the rice-based diabetes-specific formula (medical food for diabetes mellitus)

The novel rice-based MFDM was developed by SC and CH from the Department of Nutrition, Faculty of Public Health, Mahidol University and manufactured by DOD Biotech Public Company Limited (Samut Sakhon, Thailand). The ingredients of the MFDM are shown in **Table 1** and the macronutrients and caloric distribution of the MFDM are shown in **Table 2**. The available carbohydrate of the test food was analyzed by an external accredited nutrient analysis laboratory using standard methods (17). After all the ingredients were mixed, heated, and filtered, they were spray-dried, yielding the MFDM in powder form. The MFDM was developed with the most recent nutrition guidelines and recommendations in mind (18–23). Carbohydrates accounted for 43.5% of energy sources. The rice flour from white and brown rice underwent physical modification (alternated heating and cooling) to increase the proportion of slowly digestible and resistant carbohydrates (24). Fructose and isomaltulose also contributed to sources of carbohydrates. Rice bran and soluble fiber were added as sources of fiber. Sources of protein were rice-, pea-, and soy-based, and sources of fat were blends of rice bran oil and canola oil. Micronutrient mixes were added to satisfy daily requirements (21, 22). The rice-based formula was thus vegan, with no wheat, egg, milk, meat, or meat derivatives as ingredients. This formula could be particularly attractive for people with diabetes who are interested in following a plant-based diet.

**Table 1:** Ingredients of the novel rice-based medical food for diabetes (MFDM powder formula).

Ingredients	MFDM powder (% Dry weight)
Plant-based protein blends (Rice/Soy/Pea)	22.91
Modified rice flour	22.32
Fructose	11.50
Isomaltulose	15.63
Dietary fiber	4.85
Plant base oil blends (canola and rice bran oil)	16.97
Vitamin and mineral premix	5.82

MFDM, medical food for diabetes mellitus.

**Table 2:** The macronutrient composition of the MFDM, SF, and DSF.

Characteristics	Rice-based medical food for diabetes mellitus (MFDM) powder formula		Commercial product 1 Standard formula (SF) Fresubin 2 kcal Fibre		Commercial product 2 Diabetes-specific formula (DSF) Glucerna SR Triple Care	
	Usual serving size (55 g dilute to 250 ml)	Serving size in Study 2 (47.7 g dilute to 300 ml)	Usual serving size (200 ml)	Serving size in Study 2 (114.7 ml dilute to 300 ml)	Usual serving size (230 ml)	Serving size in Study 2 (287.5 ml dilute to 300 ml)
% Macronutrient distribution (carbohydrates: protein: fat)	44:17:39		45:20:35		43:20:37	
Energy (kcal)	253.0	219.4	400.0	229.4	223.0	278.8
Carbohydrates (g)	28.8	25.0	43.6	25.0	20.0	25.0
Fiber (g)	1.5	1.3	3.0	1.7	2.1	2.6
Protein (g)	12.1	10.5	20.0	11.5	10.0	12.5
Fat (g)	10.0	8.6	15.6	9.0	8.1	10.1
SFA (g)	1.7	1.5	1.2	0.7	NA	NA
MUFA (g)	4.4	3.8	11.6	6.7	Oleic acid 5.3	Oleic acid 6.6
PUFA (g)	3.2	2.8	2.8	1.6	α-Linolenic acid 0.4	α-Linolenic acid 0.5

NA, data not available; SFA, saturated fatty acids; MUFA, monounsaturated fatty acids; PUFA, polyunsaturated fatty acids. Data is shown as the amount per usual serving size and the amount per serving used in Study 2. In Study 2, serving sizes were matched so that 25 g of carbohydrates would be available from each formula.

### Study 1: Glycemic index and glycemic load in healthy individuals

#### Study design and setting

This study was designed in compliance with ISO 26642:2010 (E) (25) and was performed at the Siriraj Institute of Clinical Research (SiCRES) at the Faculty of Medicine Siriraj Hospital, Mahidol University, Bangkok. This study was prospectively registered at the Thai Clinical Trials Registry (www.thaiclinicaltrials.org, registration number TCTR20210731001) and was approved by the Human Research Protection Unit, Faculty of Medicine Siriraj Hospital, Mahidol University before the first participant was enrolled.

In this study, each participant presented to the study center for 3 study visits which included (in the order of occurrence):

- Visits 1 and 2: consuming 50 g of glucose dissolved in 300 ml of water which served as the standard food
- Visit 3: consuming MFDM which contained 50 g of glucose, prepared by dissolving 95.42 g of MFDM powder in water to reconstitute a 300 mL solution

All participants had the same study visit sequences since the interventions were unlikely to have any period or order effects. The washout period used was between 1 to 30 days, since it was unlikely that the effects of the interventions would be carried over beyond one day (25). The interventions were administered in an unblinded manner.

#### Participants

Inclusion criteria included age 18-60 years, BMI 18.5-23 kg/m^2^, fasting plasma glucose<100 mg/dL, no prior history of diabetes or prior use of glucose-lowering drugs, no allergy to study product or its constituents, and the ability to read and write in the local language (Thai). Exclusion criteria included serious illness requiring medical attention (including but not limited to cancer, cardiovascular, respiratory, renal, or hepatic diseases) within 1 year of study participation, any surgery or hospital admission within 3 months of study participation, use of thyroid hormones or medications which can affect plasma glucose levels (including steroids, antipsychotic drugs, or protease inhibitors), history of malabsorptive disorders such as inflammatory bowel disease or previous bowel resection, pregnancy, and lactation. Withdrawal criteria included allergic symptoms to study products, and participants’ refusal to continue in the study.

#### Interventions

Participants were recruited from online advertisements and billboards. After an initial telephone screening interview, eligible participants presented to the study center for screening blood tests which included fasting plasma glucose and HbA1c. These tests were measured from the venous blood by the clinical pathology laboratory at the Faculty of Medicine Siriraj Hospital, Mahidol University, Bangkok, Thailand (Cobas 8000 Analyzer Series, Roche Diagnostics, Indianapolis, United States). HbA1c was measured according to the standards of the National Glycohemoglobin Standardization Program (NGSP). Those who met all the inclusion criteria were enrolled in the study. Subjects were asked to fast after 8 pm and avoid exercises before attending each study visit.

At the first study visit, participants’ baseline data including sex, age, body weight, body mass index, waist circumference, hip circumference, blood pressure, medical history, and current medications were collected. Height was measured using a calibrated stadiometer. Body weight (BW) was measured, using a calibrated weighing scale (TANITA BC-587, Tanita Corporation, Tokyo, Japan). Body mass index (BMI) was calculated by BW (kg)/(height [m]^2^). Waist circumference (WC) and hip circumference (HC) were measured using non-stretchable tape. WC was measured midway between the iliac crests and the lowest ribs. HC was measured at the widest protrusion of the buttocks. The waist-to hip ratio (W/H ratio) was calculated by WC/HC. At the first visit, blood was drawn for baseline biochemical testing including insulin, cholesterol, triglyceride, HDL cholesterol, liver function tests, serum creatinine, and high-sensitivity CRP (Cobas 8000 Analyzer Series, Roche Diagnostics, Indianapolis, United States). LDL-c levels were calculated by the Friedewald formula.

Participants were asked to consume the glucose solution or MFDM at an even pace and to finish within 15 mins. After finishing, drinking up to 250 ml of water was allowed. For each subject, a blood sample was taken in the fasting state and used as the baseline blood glucose concentration. After consumption of the reference food or test food, blood samples were collected at 15, 30, 45, 60, 90, and 120 mins, and assayed for glucose. Precautions were taken during the screening, enrollment, and intervention periods to minimize the risk of SARS-CoV-2 transmission including avoiding crowding, determining a history of high-risk exposure, and asking participants to perform SARS-CoV-2 rapid testing before coming in for study visits.

#### Sample size

At least ten participants were included in the study to comply with the ISO 26642:2010(E) standards. A participant may be considered an outlier if the glycemic index measured from that participant was 2 standard deviations below or above the mean of the group of 10 participants or more. Outliers may be excluded if at least 10 participants remain available for analysis.

#### Outcomes and statistical analysis

The area under the curve (AUC) of glucose was calculated using the trapezoidal rule. Only positive areas under the curve were considered. The glycemic index and glycemic load for each participant were calculated as follows:

$Glycemic\;index\;(GI)=\frac{AUC\;of\;50g\;carbohydrates\;test\;food}{AUC\;of\;50g\;carbohydrates\;reference\;food}\times 100$

$Glycemic\;load\;(GL)=\frac{carbohydrate\;content\;(g)\times Glycemic\;index}{100}$

The glycemic index and glycemic load of each test product were reported as the mean ± standard error of the mean (SEM).

### Study 2: Glycemic, insulin, and gastrointestinal hormone responses in adults with diabetes or at risk for diabetes

#### Study design and setting

The study was designed as a four-arm randomized double-blinded crossover clinical trial. This trial was prospectively registered at the Thai Clinical Trials Registry (www.thaiclinicaltrials.org, registration number TCTR20210730007) and was approved by the Human Research Protection Unit, Faculty of Medicine Siriraj Hospital, Mahidol University before study initiation. The study was performed at the Siriraj Institute of Clinical Research (SiCRES) at the Faculty of Medicine Siriraj Hospital, Mahidol University, Bangkok, Thailand. We chose this study design, in which each participant received all interventions and acted as his/her own control, to maximize statistical power and to allow comparison of data at both the individual and group levels. The four interventions in this study included the consumption of one of the following products:

A. Rice-based diabetes-specific formula, powder (MFDM)

B. Rice-based diabetes-specific formula, liquid (MFDM-liquid)

C. Commercially available standard non-diabetes formula (SF)

D. Commercially available diabetes-specific formula (DSF)

This report focuses on the data from the MFDM, SF, and DSF interventions. Data from the MFDM-liquid intervention will be published in a future report.

These interventions were chosen to allow for the comparison of the MFDM with either a commercially available diabetes-specific formula or a standard, non-diabetes-specific formula, with an assumption that the MFDM would elicit glycemic and hormonal responses similar to the commercially available DSF, but would elicit less glycemic responses and more favorable hormonal responses compared with commercially available SF. Each intervention was separated by a washout period of between 7 to 14 days, because the effects of study products on the primary outcome, postprandial glucose response, were unlikely to carry over beyond such a period (26, 27). There was no significant change to the study design after trial commencement.

#### Randomization and blinding

The participants were randomized to receive interventions in one of the following orders 1) ABCD, 2) BCDA, 3) CDAB, and 4) DABC, in a ratio of 1:1:1:1 using randomization with a permuted block of four. Study personnel (pharmacists) who were not involved in subject enrollment or data collection performed randomization and blinding. All other personnel and all participants were blinded to the order of the intervention. The pharmacists utilized an online program (sealedenvelope.com) to generate a randomization list (28) which was not disclosed to other study personnel. At each study visit, the pharmacists prepared study products according to the randomization list which was served to participants in a similar manner at all visits (in liquid form with the same volume, using the same large cups). Apart from the different study products which were served, all other interventions at all study visits were the same.

#### Participants

Inclusion criteria included age 18-60 years, BMI 25-35 kg/m^2^, a diagnosis of either 1) prediabetes (HbA1c 5.7% - 6.4% or FPG 100-125 mg/dL or oral glucose tolerance test 140-199 mg/dL) or 2) recent-onset diabetes of no more than 6 years made by HbA1c, fasting plasma glucose, or random plasma glucose according to the most recent American Diabetes Association Standards of Medical Care (29). For participants with type 2 diabetes, only those who were managed with lifestyle modification alone without the use of glucose-lowering drugs were enrolled. Also, only those who had received at least 2 doses of vaccines against SARS-CoV-2 were considered for enrollment. Exclusion criteria included the use of any medications which can affect plasma glucose levels within 3 months of study initiation, any prior insulin use, gastroparesis, HbA1c above 9%, blood pressure ≥ 180/110 mmHg, triglyceride levels above 500 mg/dL, severe hypoglycemia or hyperglycemia requiring hospital admission within 1 year of study initiation, any severe illnesses or surgeries within 2 weeks of study initiation, known allergy to study products or their components, weight loss or weight gain of 5 kg or more within 6 weeks of study initiation, history of bariatric surgery, history of malabsorptive disorders such as inflammatory bowel diseases or short bowel syndrome, pregnancy, and lactation. Withdrawal criteria included allergic reactions to study products, requests from subjects to withdraw, and worsening glycemic control during the study requiring the use of glucose-lowering agents or insulin.

#### Formulas

The comparison of composition between the MFDM, SF, and DSF formulas is shown in **Table 2**. The SF and DSF formulas were chosen to best match the macronutrient distribution of the MFDM formula (**Table 2**). The serving sizes of all formulas used in Study 2 were matched in terms of carbohydrate amounts, with each serving containing 25 g of carbohydrates, as follows:

- Standard non-diabetes formula (SF, commercial formula 1, Fresubin 2 kcal Fibre, Fresenius Kabi, Bad Homburg, Germany): usual serving size = 200 ml, serving size in Study 2 = 114.7 ml diluted to 300 ml
- Diabetes-specific formula (DSF, commercial formula 2, Glucerna SR Triple Care, Abbott Laboratories, Chicago, Illinois, United States): usual serving size = 230 ml, serving size in Study 2 = 287.5 ml diluted to 300 ml
- Rice-based diabetes-specific formula, powder (MFDM): usual serving size = 55g diluted to 250 ml, serving size in Study 2 = 47.7 g diluted to 300 ml

The amount of carbohydrates of 25g was chosen to allow serving sizes to be reasonable and not too large for a single meal. We chose to administer the formulas orally instead of through a feeding tube since both methods would likely induce similar physiological responses and oral administration was more comfortable for the study participants.

#### Interventions

Participants were recruited from posters and online advertisements. After a screening phone interview, eligible participants were selected and asked to visit the study center for screening blood tests (fasting plasma glucose and HbA1c). Those who met all the inclusion criteria were enrolled in the study. Subjects were asked to fast after 8 pm and avoid exercises before attending each study visit.

At the first study visit, participants’ baseline data including sex, age, body weight, body mass index, waist circumference, hip circumference, blood pressure, medical history, and current medications were collected similarly as in Study 1. All products were diluted to the same final volume of 300 ml. Participants were asked to consume study products at an even pace within 15 mins with up to 250 ml of water afterward. Blood samples were collected at baseline and 15, 30, 45, 60, 90, and 120 mins after consuming study products. Blood samples were assayed for glucose, insulin, and gastrointestinal hormones as discussed below. Satiety and hunger were assessed at each time point using visual analog scales (VAS). Similar precautions were taken as in Study 1 to reduce the risk of SARS-CoV-2 transmission during the study.

#### Hunger and satiety

A visual analog scale (VAS) was used to assess hunger and satiety at baseline, and then at 15, 30, 45, 60, 90, 120, and 180 mins after consuming study products. The participants self-reported their hunger and satiety using the VAS which comprises ten 1-centimeter segments, representing a score from 0 to 10. All volunteers were asked not to discuss their feelings or ratings with their counterparts.

#### Gastrointestinal hormones

Gastrointestinal hormones, including active ghrelin, glucose-dependent insulinotropic peptide (GIP), active glucagon-like peptide-1 (GLP-1), and peptide YY (PYY) were measured using a bead-based multiplex assay kit (MILLIPLEX^®^ Multiplex Assays ELISA kit number HMHEMAG-34K, Luminex, Austin, TX, United States) at baseline and 15, 30, 45, 60, 90, 120 and 180 mins during the intervention period. Samples were assayed in duplicates using a multiplex analyzer (Luminex 200, Luminex, Austin, TX, United States) and data analyses were conducted using xPONENT software (Luminex, Austin, TX, United States).

#### Sample size

The sample size was calculated using nQuery Advisor version 6.01 (Statsols (Statistical Solutions Ltd), Cork, Ireland). Previously, Garcia-Rodriguez et al. demonstrated that the mean ± SEM of AUC of glucose in participants with type 2 diabetes after consuming Glucerna SR was 415 ± 71 mmol/l*min (10). Accounting for within-participant variability in glycemic responses, to demonstrate the difference of 100 mmol/l*min of the novel diabetes-specific formulas against Glucerna SR with a two-sided test significance level of 0.05 and a power of 80%, at least 27 participants were required.

#### Outcomes and statistical analysis

There was no significant change to trial outcomes after trial commencement. Characteristics of participants at baseline were described using standard descriptive statistics. Data were presented as the median and interquartile range (IQR). The area under the curve (AUC) of glucose, insulin, other gastrointestinal hormones, and satiety or hunger visual analog scale (VAS) score were calculated using the trapezoidal rule. Only positive areas under the curve above the baseline value were considered, except for ghrelin for which only negative areas under the baseline were considered. AUC of glucose, insulin and other gastrointestinal hormones were compared between interventions using paired *t*-tests for pairwise comparisons. No adjustments for multiplicity were applied during analyses in this study. Statistical analyses were performed using IBM SPSS Statistics for Windows, version 18.0 (IBM Corp., Armonk, NY, USA), GraphPad Prism version 8.4.3 (GraphPad Software, San Diego, CA, United States), and Microsoft Excel 2019 (Microsoft Corporation, Redmond, WA, USA).

## Results

### Study 1: Glycemic index and glycemic load in healthy individuals

**Figure 2:**
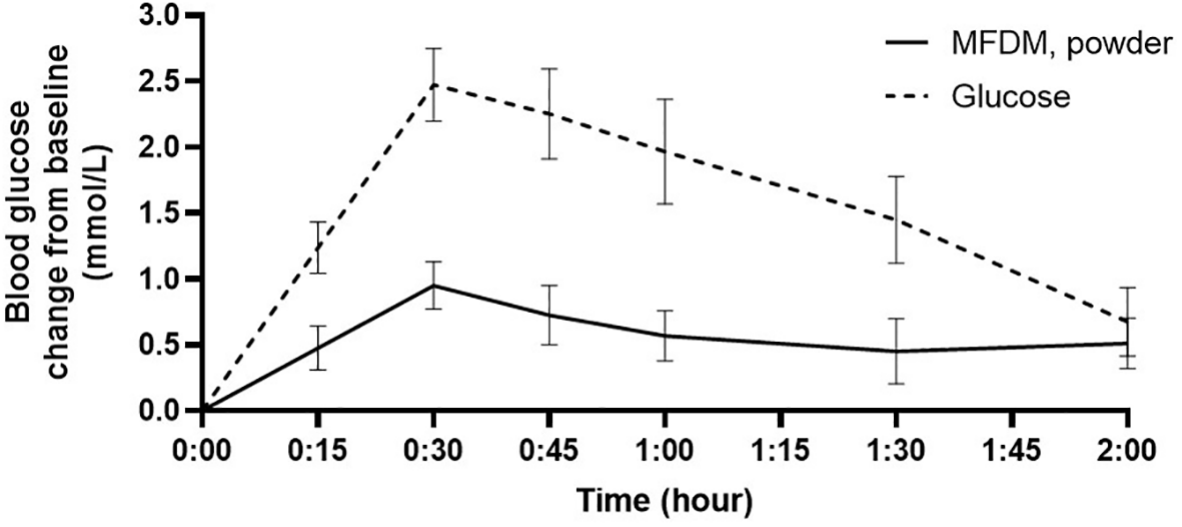
Glycemic response in healthy subjects to MFDM powder formula compared with glucose in Study 1. Error bars represent SEM.

The participant flow is illustrated in **Figure 1**. Screening and enrollment of subjects started in November 2021, and all study procedures were completed before October 2022. Out of 11 subjects enrolled, one was excluded as an outlier (measured GI was beyond 2 standard deviations from group mean), leaving 10 subjects in the final analysis. The baseline characteristics of subjects are shown in **Table 3**. The glycemic response to the MFDM powder formula had a gradual rise before reaching its peak at 30 minutes and was less pronounced compared with glucose (**Figure 2**). The calculated glycemic index and glycemic load of the MFDM powder formula were summarized in **Table 4**. All participants tolerated the formula well without any adverse events.

**Figre 1:**
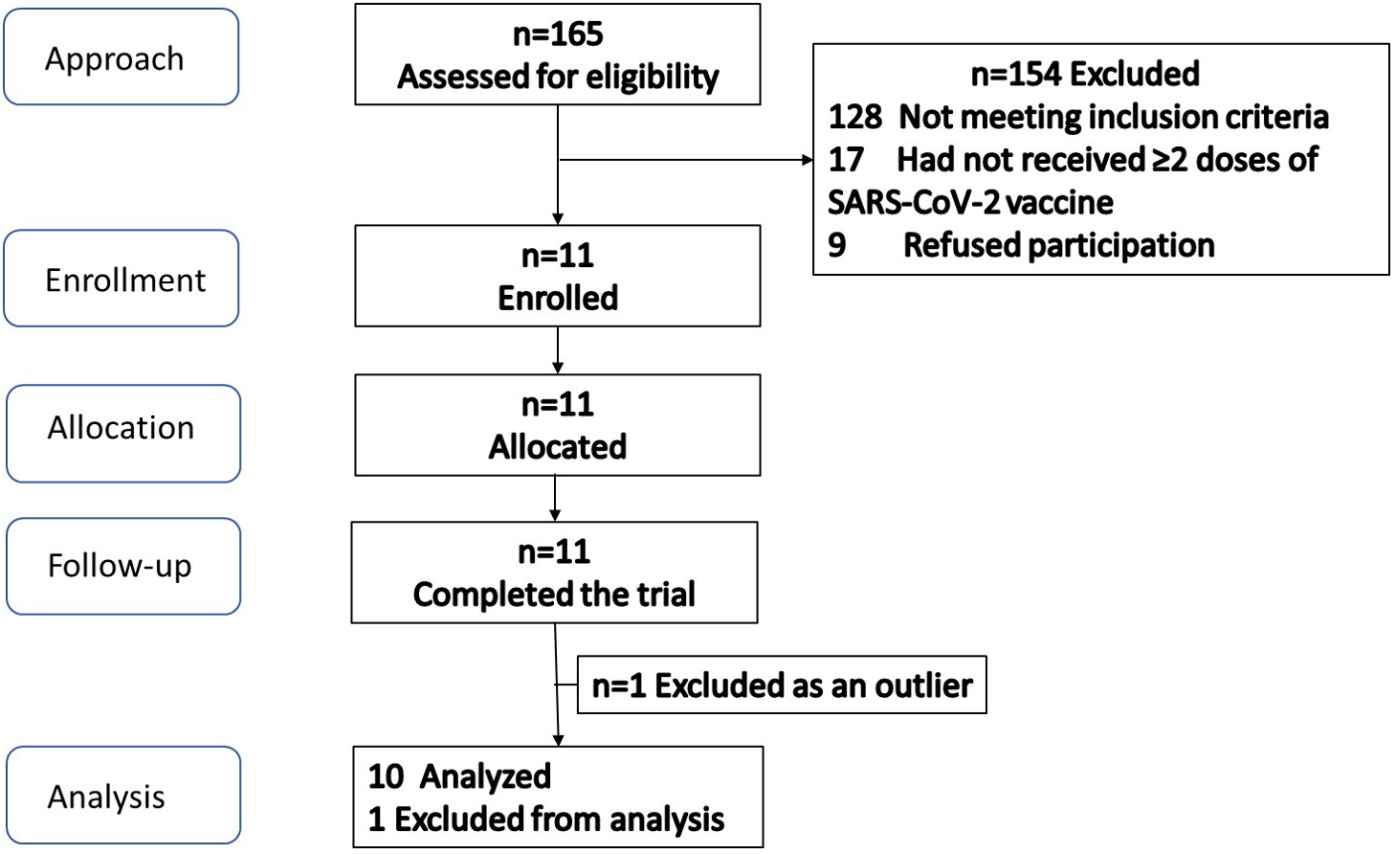
Participant flow diagram for Study 1.

**Table 3:** Baseline characteristics of healthy subjects in Study 1. Data are presented as the median and interquartile range (IQR) unless noted otherwise.

Characteristics	Healthy Subjects (n = 10)
Male (n, %)	4 (40%)
Age (years)	32.0 (28.0, 44.0)
Height (cm)	162 (155.8, 170.0)
Weight (kg)	54.5 (49.3, 61.7)
BMI (kg/m^2^)	20.8 (19.5, 22.3)
Waist (cm)	76.0 (72.8, 78.3)
Hip (cm)	84.5 (81.0, 88.8)
Waist/Hip ratio	0.88 (0.85, 0.91)
Systolic Blood Pressure (mmHg)	114.0 (100.8, 122.8)
Diastolic Blood Pressure (mmHg)	65.5 (64.0, 84.0)
Pulse Rate (bpm)	76.0 (64.0, 84.0)
Body Fat (%)	20.6 (17.4, 28.8)
HbA1c (%)	5.3 (5.1, 5.4)
Fasting Plasma Glucose (mg/dL)	78.0 (74.8, 82.2)
Insulin (uU/ml)	4.8 (2.3, 7.6)
Creatinine (mg/dL)	0.76 (0.67, 0.87)
AST (SGOT) (U/l)	18.0 (15.0, 20.3)
ALT (SGPT) (U/l)	12.0 (10.5, 13.3)
Cholesterol (mg/dL)	173.5 (155.3, 205.3)
HDL (mg/dL)	56.5 (43.0, 68.0)
LDL (mg/dL)	100.7 (90.2, 131.6)
Triglyceride (mg/dL)	61.5 (49.3, 100.5)

**Table 4:** Measured glycemic index and glycemic load of the novel rice-based medical food for diabetes mellitus (MFDM) powder formula.

Measured parameters	Medical food for diabetes mellitus (MFDM) powder
Glycemic index (mean ± SEM)	39 ± 6
Glycemic index category**	Low
Glycemic load* (mean ± SEM)	11 ± 2
Glycemic load category***	Medium

*Calculated based on available carbohydrates per serving; MFDM contained 28.82 g of carbohydrates per serving (55g).

**Glycemic index is considered low if ≤ 55, medium if >55 and ≤70, and high if >70.

***Glycemic load is considered low if ≤ 10, medium if 11-19, and high if ≥20.

### Study 2: Glycemic, insulin, and gastrointestinal hormone responses in adults with diabetes or at risk for diabetes

**Figure 3:**
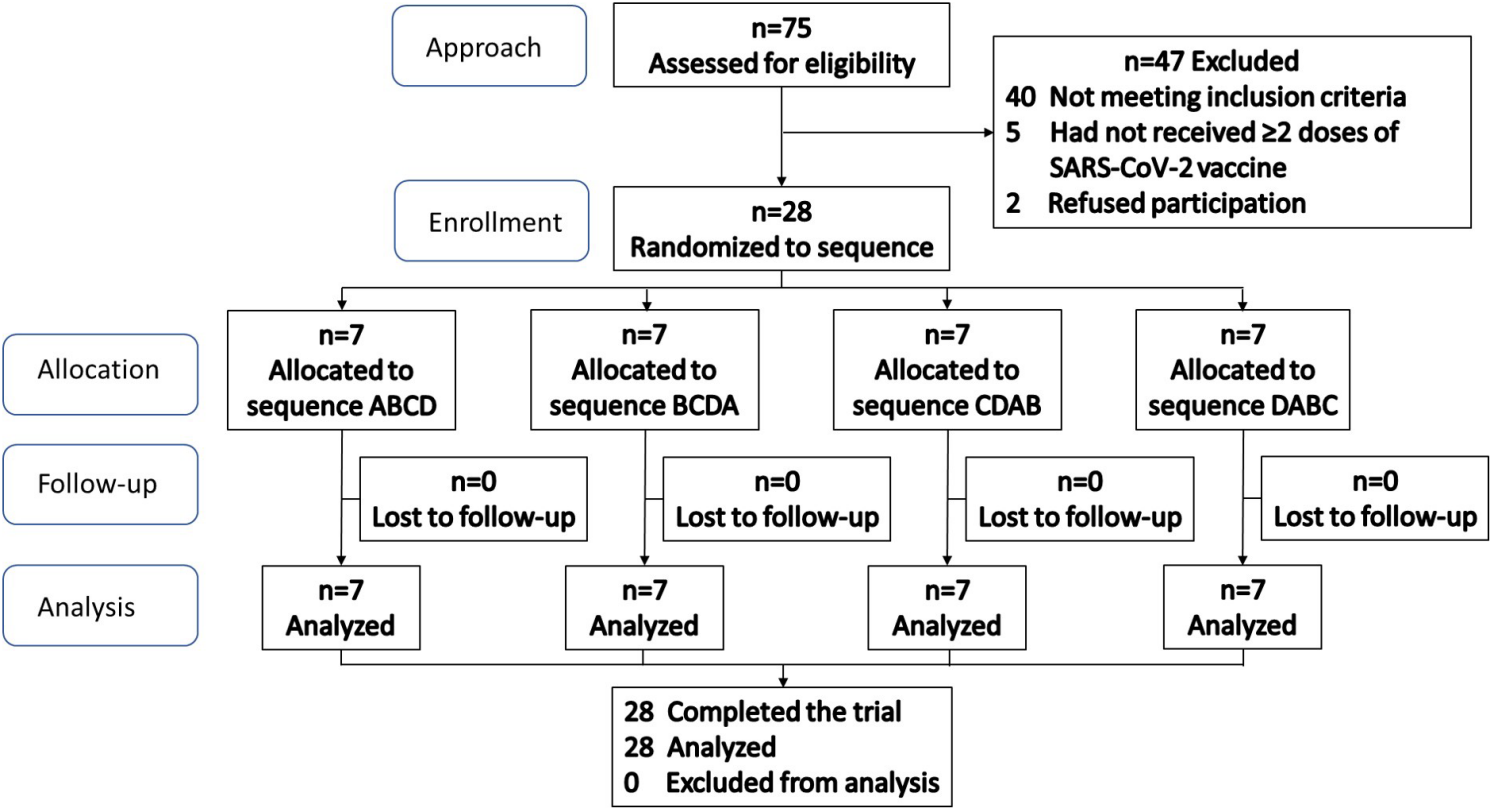
Participant flow diagram for Study 2.

The participant flow diagram is shown in **Figure 3**. Screening and enrollment of subjects started in November 2021, and all study procedures were completed before October 2022. After randomization, no subjects were lost or excluded, and data from all subjects were analyzed. The trial ended after the completion of all procedures. The baseline characteristics of all participants are shown in **Table 5**.

**Table 5:** Baseline characteristics of participants in Study 2. Data are shown as the median and interquartile range (IQR) unless noted otherwise.

Characteristics	Total (n=28)	Sequence			
		ABCD (n=7)	BCDA (n=7)	CDAB (n=7)	DABC (n=7)
Diabetes (n, %)	13 (46.4%)	4 (57.1%)	4 (57.1%)	2 (22.2%)	3 (42.9%)
Male (n, %)	9 (32.1%)	2 (22.2%)	3 (42.9%)	2 (22.2%)	2 (22.2%)
Age (years)	43.0 (37.5, 51.0)	47.0 (37.0, 58.0)	41.0 (35.0, 46.0)	44.0 (39.0, 46.0)	42.0 (36.0, 57.0)
Height (cm)	163.0 (157.3, 166.8)	161.0 (158.0, 165.0)	165.0 (155.0, 175.0)	163.0 (157.0, 166.0)	160.0 (154.0, 167.0)
Weight (kg)	79.2 (71.6, 89.5)	77.3 (68.9, 85.3)	80.7 (74.4, 97.6)	85.6 (68.3, 86.4)	73.1 (66.6, 97.8)
BMI (kg/m^2^)	30.3 (28.3, 32.2)	30.5 (28.3, 32.5)	30.0 (29.6, 32.6)	31.4 (27.6, 32.3)	30.1 (26.9, 32.0)
Waist circumference	101.5 (97.0, 108.0)	99.0 (87.0, 117.0)	104.0 (101.0, 113.0)	99.0 (93.0, 104.0)	102.0 (93.0, 108.0)
Hip circumference	111.0 (104.0, 115.8)	112.0 (97.0, 119.0)	112.0 (108.0, 115.0)	110.0 (105.0, 117.0)	107.0 (102.0, 115.0)
Waist-hip ratio	0.92 (0.89, 0.96)	0.93 (0.88, 0.98)	0.94 (0.93, 0.99)	0.89 (0.88, 0.93)	0.91 (0.89, 1.02)
Systolic blood pressure (mmHg)	135.0 (124.8, 144.3)	134.0 (117.0, 145.0)	139.0 (133.0, 148.0)	136.0 (122.0, 150.0)	134.0 (132.0, 142.0)
Diastolic blood pressure (mmHg)	83.0 (69.3, 86.8)	68.0 (62.0, 92.0)	82.0 (78.0, 85.0)	84.0 (73.0, 85.0)	86.0 (68.0, 97.0)
Pulse (bpm)	76.0 (68.0, 80.0)	68.0 (64.0, 80.0)	80.0 (76.0, 80.0)	73.0 (60.0, 78.0)	74.0 (70.0, 98.0)
Body fat (%)	38.9 (31.9, 43.1)	38.9 (32.8, 43.2)	38.9 (31.3,42.3)	39.9 (27.6, 43.5)	37.9 (33.2, 42.8)
Laboratory investigations					
HbA1c (%)	6.2 (5.7, 6.9)	6.4 (5.9, 7.6)	6.8 (5.7, 6.9)	5.6 (5.3, 6.5)	6.2 (6.1, 6.6)
Fasting plasma glucose (mg/dL)	104.5 (87.0, 115.8)	105.0 (93.0, 138.0)	109.0 (92.0, 115.0)	87.0 (82.0, 116.0)	104.0 (85.0, 120.0)
Insulin (uU/mL)	18.1 (11.9, 23.5)	18.1 (11.9, 19.7)	15.4 (13.9, 21.2)	15.7 (8.8, 29.9)	22.5 (13.4, 25.1)
hsCRP (mg/L)	1.9 (1.0, 6.1)	3.6 (1.1, 6.8)	1.7 (1.1, 8.2)	1.0 (0.5, 2.2)	3.6 (1.8, 9.9)
Creatinine (mg/dL)	0.67 (0.63, 0.85)	0.66 (0.63, 0.83)	0.75 (0.63, 0.87)	0.66 (0.64, 0.81)	0.70 (0.61, 1.00)
AST (U/L)	18.5 (15.0, 30.8)	15.0 (14.0, 22.0)	22.0 (15.0, 37.0)	18.0 (15.0, 55.0)	19.0 (15.0, 31.0)
ALT (U/L)	19.0 (14.0, 49.8)	14.0 (13.0, 31.0)	19.0 (14.0, 52.0)	22.0 (18.0, 96.0)	19.0 (9.0, 59.0)
Alkaline phosphatase (U/L)	61.5 (55.0, 76.5)	65.0 (63.0, 71.0)	77.0 (43.0, 88.0)	55.0 (50.0, 57.0)	61.0 (55.0, 84.0)
Cholesterol (mg/dL)	175.0 (146.0, 200.8)	175.0 (141.0, 200.0)	174.0 (144.0, 189.0)	175.0 (134.0, 192.0)	219.0 (160.0, 224.0)
HDL cholesterol (mg/dL)	42.5 (35.3, 51.8)	43.0 (35.0, 52.0)	42.0 (36.0, 57.0)	34.0 (34.0, 47.0)	48.0 (41.0, 54.0)
LDL cholesterol (mg/dL)	96.2 (81.0, 124.1)	115.0 (66.4, 122.0)	85.2 (67.0, 115.2)	109.0 (82.6, 124.8)	98.2 (80.8, 144.2)
Triglyceride (mg/dL)	129.0 (82.0, 211.5)	104.0 (85.0, 173.0)	139.0 (75.0, 242.0)	116.0 (81.0, 129.0)	139.0 (71.0, 302.0)

### Glucose and insulin responses

**Figure 4:**
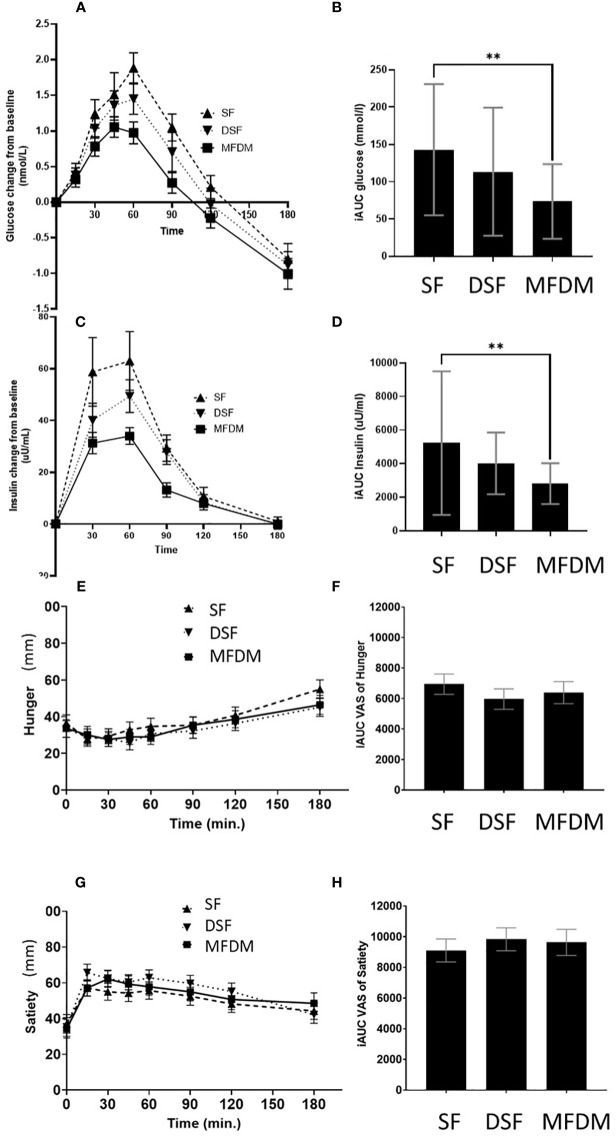
Response curves and AUC of glycemic responses **(A, B)**, insulin responses **(C, D)**, hunger **(E, F)**, and satiety **(G, H)** from Study 2. SF standard formula (– – –), DSF diabetes-specific formula (•••), MFDM rice-based medical food for diabetes (**—**), ** denotes *p*-value<0.01.

The MFDM powder formula elicited significantly lower glycemic response compared with SF (*p*<0.01), however, the responses were not significantly different when compared with DSF (**Figures 4A, B**). In terms of insulin responses, the MFDM powder formula also resulted in significantly lower insulin responses compared with SF (*p*<0.01) but did not significantly differ from DSF (**Figures 4C, D**).

### Hunger

The MFDM formula suppressed hunger over the course of 180 minutes (**Figures 4E, F**). However, there was no statistically significant difference between the MFDM and SF or DSF.

### Satiety

The MFDM formula promoted satiety over the course of 180 minutes (**Figures 4G, H**). However, no statistically significant difference was observed between the MFDM and SF or DSF.

### Gastrointestinal hormone responses

**Figure 5:**
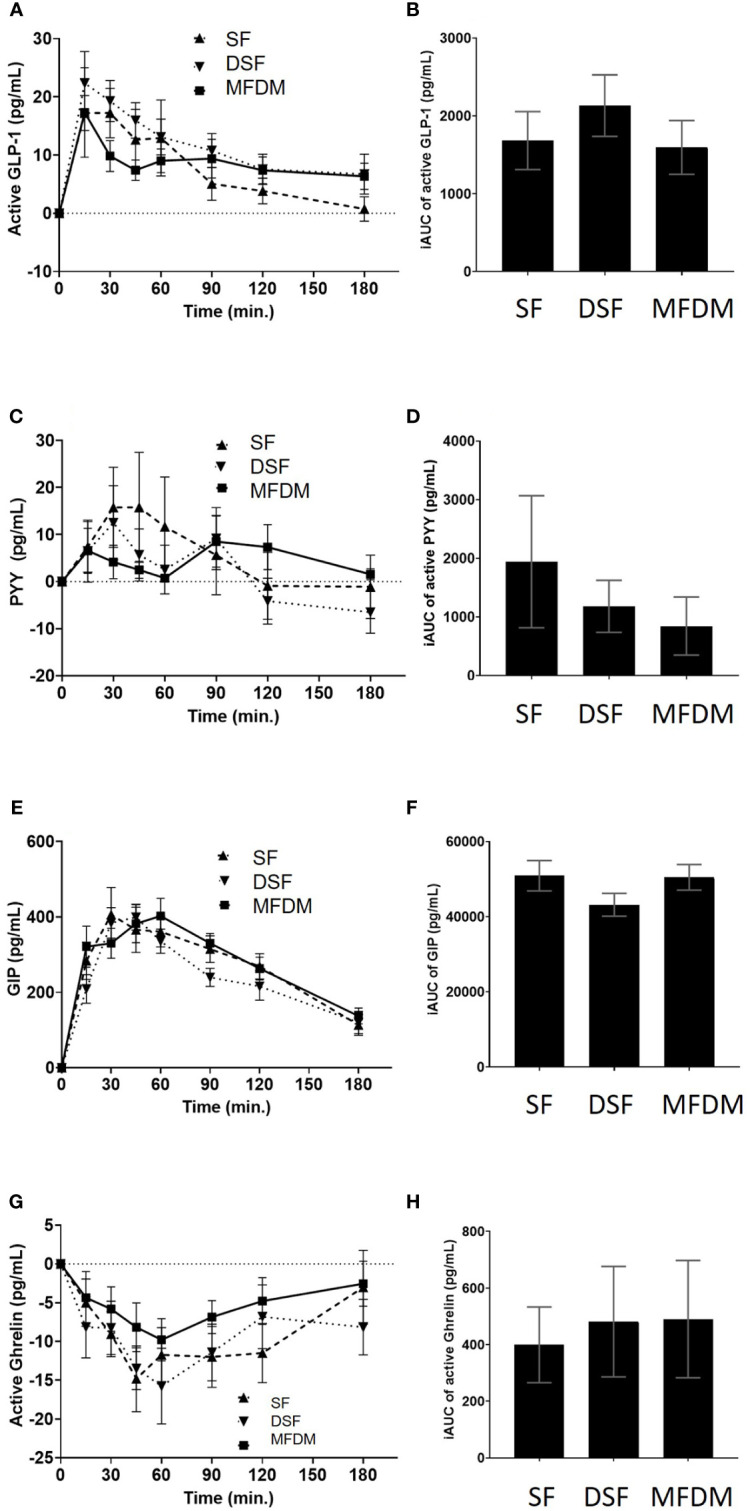
Response curves and AUC of active GLP-1 **(A B)**, PYY **(C D)**, GIP **(E, F)**, and active ghrelin **(G, H)** from Study 2. SF standard formula (**- - -**), DSF diabetes-specific formula (•••), MFDM rice-based medical food for diabetes (**—**).

After consumption of the MFDM powder formula, stimulation of active GLP-1, PYY, and GIP and suppression of ghrelin were noted (**Figures 5A–H**). However, these changes in gastrointestinal hormones did not differ when compared with either SF or DSF.

### Tolerability and adverse events

There was no adverse events or side effects during the study. All participants tolerated the new MFDM powder formula well without any gastrointestinal side effects.

## Discussion

In the first study, we demonstrated that the novel rice-based MFDM powder formula had attenuated postprandial glycemic response compared with glucose in a healthy population, resulting in a low GI and a low-to-intermediate GL. In the second study performed in adults with diabetes or at risk for diabetes, when compared with the commercially available standard formula (SF), the MFDM powder formula had significantly lower postprandial glycemic and insulin responses even when the serving sizes were matched for the carbohydrate content and macronutrient distribution (**Table 1**). Multiple studies have previously shown that diabetes-specific enteral formulas can reduce postprandial hyperglycemia in the populations at risk (26, 27, 30). Our findings confirmed the potential use of the MFDM powder formula as a diabetes-specific formula that can reduce the risk of postprandial hyperglycemia when used in patients who are at risk for postprandial hyperglycemia and its associated morbidity. The physical modification of the rice starch using temperature and the combination of fiber, protein, and fat of the MFDM formula likely explained these findings.

We also demonstrated in the second study that the MFDM powder formula had similar glucose and insulin responses when compared with a commercially available diabetes-specific formula (DSF) in adults at risk for or diagnosed with early diabetes. The carbohydrate sources differed significantly between the two formulas. Commercial product 2 contained maltodextrin (40%), Fibersol 2 (25%), and sucromalt (20%), while the MFDM powder formula contained physically modified rice flour (22.3%), fructose (11.5%), and isomaltulose (15.6%) (**Table 1**). Rice flour is readily available and provides an important source of carbohydrates and protein in large parts of the world (31). We have demonstrated here that rice flour can be physically modified using temperature to increase the amount of slowly digestible carbohydrates (24) and can be effectively used as the main ingredient of a medical food suitable for patients with diabetes or at risk for hyperglycemia. Although attempts were made to best match the carbohydrate and macronutrient distribution among the three test formulas in Study 2, it must be noted that in the serving sizes used, the commercial product 2 provided greater energy (278.8 vs 219.4 kcal), protein (12.5 g vs 10.5 g), fat (10.1 g vs 8.6 g), and fiber (2.6 vs 1.3 g) compared with the MFDM powder formula (**Table 2**). The increased amount of protein and fiber from commercial product 2 could have greater stimulatory effects on insulin release (9), while greater fat content could have slowed gastric emptying (9). Both effects may have reduced glycemic responses after the participants consumed commercial product 2 in this study. However, these hypotheses have to be fully tested in future studies.

In Study 2 we showed that the novel MFDM powder formula produced suppressed hunger and maintained satiety throughout the course of each intervention day. The formula also stimulated the release of active GLP-1, GIP, and PYY, and suppressed active ghrelin over the course of 180 minutes. However, such effects on hunger, satiety and postprandial gastrointestinal hormones did not differ significantly from the commercially available SF and DSF. Some previous studies demonstrated the ability of diabetes-specific formulas to stimulate greater postprandial GLP-1 release compared with control foods or standard formulas (11, 26, 30). However, other studies were not able to show such differences (10). The heterogeneous nature of these studies in terms of the formulas used, interventions, and participant characteristics made it very difficult to compare results across studies. The fact that diabetes impairs incretin function by attenuating GLP-1 and GIP responses (32) also likely contributed further to heterogeneity between studies, where some of which enrolled participants at early stages of diabetes, while others included those who were further along in the course of their disease. It has been shown that chronic consumption of resistant starch has the potential to increase GLP-1 release and modulate the gut microbiota (33). Although the MFDM powder formula contained a significant amount of resistant starch, studies with longer duration are required to assess the long-term effects of the MFDM formula on GLP-1 releases and the microbiome in people with diabetes, such as bedridden patients who cannot eat and require long-term enteral nutrition.

The limitations of our studies included a short-term study design. Although we were able to assess postprandial glycemic and hormonal responses after consumption of the novel rice-based MFDM powder formula, long-term studies are required to investigate the effects of the MFDM formula on glycemic control, body weight, and other health outcomes over a longer period. The generalizability of our study may also be limited as the study was single-center and enrolled only healthy individuals, those at risk for diabetes, and those with early-onset type 2 diabetes. The effects of the MFDM powder on people with long-standing diabetes must be assessed in another study. The strengths of our studies, primarily Study 2, included a double-blinded multi-arm randomized crossover design, and matching of macronutrient distribution and carbohydrate content per serving in the three formulas tested.

To conclude, we showed in this study that in healthy participants, the rice-based MFDM powder formula had a low glycemic index and a low-to-intermediate glycemic load. We also showed that in adults at risk for diabetes or with early type 2 diabetes, the MFDM powder formula produced significantly less glucose and insulin responses compared with a commercially available standard formula (SF). The MFDM powder formula may be an option for patients who require diabetes-specific formulas, particularly those who are vegan as all of its ingredients are plant-based. The use of locally sourced ingredients has lowered the cost of the MFDM to 18-24 THB (0.5-0.7 USD) per 250 ml serving compared with 70-170 THB (2-5 USD) per 250 ml serving for other commercial formulas. This likely would improve patient access and reduce the barrier to care in resource-limited settings such as in Thailand.

## Data availability statement

The original contributions presented in the study are included in the article/supplementary material. Further inquiries can be directed to the corresponding author.

## Ethics statement

The studies involving human participants were reviewed and approved by Human Research Protection Unit, Faculty of Medicine Siriraj Hospital, Mahidol University. The patients/participants provided their written informed consent to participate in this study.

## Author contributions

SC and CH developed and oversaw the production of the MFDM powder formula. The study was conceived and designed by PP, SC, and CH. NKe, CC, NKh, WD, PW, TS, and PP enrolled participants and conducted the study visits. NKe and CC performed measurements of the gastrointestinal hormones. NKe, CC, and PP performed the statistical analysis and wrote the first draft of the manuscript. All authors contributed to the manuscript revision and approved the submitted version.
